# Seismology’s acoustic debt: Robert Mallet, Chladni’s figures, and the Victorian science of earthquakes

**DOI:** 10.1080/20551940.2019.1678313

**Published:** 2019-11-05

**Authors:** Edward J. Gillin

**Affiliations:** Faculty of Music, University of Cambridge, Cambridge, UK

**Keywords:** Earthquakes, Robert Mallet, Charles Darwin, acoustics, Chladni, Victorian

## Abstract

In the nineteenth century, Ernst Chladni’s acoustic figures provided productive new experimental techniques for investigating natural phenomena. The movement of invisible forces, such as light, electricity, magnetism, and heat, were hard for natural philosophers to examine. But Chladni’s use of vibrating glass plates and sand to reveal the wave motions of sound offered an experimental framework through which to make natural phenomena visible. In Britain, it was Michael Faraday and Charles Wheatstone in the 1820s and 1830s who made best use of these practices and apparatus. Sound waves also provided new ways of thinking about earthquakes and seismic phenomena. This article explores how Robert Mallet, the first self-styled “seismologist”, examined earthquakes, drawing on broader philosophical work surrounding vibrations and acoustic waves. Mallet was keen to draw parallels between the movement of seismic shock waves and the movement of musical sounds, including those from a piano moving through a room. He was not alone in this respect. Charles Darwin, among others, noticed comparisons between the sonorous and the seismic. By contextualising Victorian seismology within the context of Victorian acoustic science, this article argues that the two disciplines were deeply connected.

It is well observed that the investigation of sound has, historically, provided valuable analogies for wider interpretations of natural phenomena.^1^ William Whewell (1794–1866), philosopher of science and inventor of the term “scientist”, described sound as one of the earliest understood forces in his influential *History of the Inductive Sciences* (1837). The science of acoustics was, he claimed, built “upon acknowledge truths”, rather than moments of long-awaited “great discovery”, such as was the case with gravity (Whewell 1857, 237). Unlike heat, light, magnetism, and electricity, sonorous waves had been measurable in terms of frequency since Pythagoras’ assertion that the musical notes of vibrating strings could be conceived of as mathematical ratios. As Whewell explained, the human ear was astonishing at detecting the musical relations between different notes, which could be “reduced to numerical relations” (Whewell 1847, 327). The relationship between pitch and number was knowable in a way that set sound apart from other invisible forces. During the late eighteenth century, Ernst Chladni’s (1756–1827) acoustic figures made the oscillations of different sonorous vibrations visible to the human eye: sound waves could in this way, Chladni argued, be seen. Unsurprisingly, such understandings of the sonorous informed new interpretations of other natural phenomena. If sound was an impulse, then heat, light, and electricity might also travel in waves. As histories of science have shown, descriptions of light were frequently made in terms of its similarity or difference to sound.^2^ This was particularly evident during the early nineteenth century in the works of John Herschel (1792–1871), Mary Somerville (1780–1872), and Whewell, who sought to make knowledge of the natural sciences accessible to new reading audiences.^3^

Yet experiments on the nature of sound not only provided resources for the examination of other natural forces. They also helped to fashion empirical frameworks for the study of the earth itself, specifically in terms of seismic activity. This article explores the evolution and adaptation of a series of experiments, conceived of for better understanding acoustic phenomena, to the examination of earthquakes in 1840s’ Britain. The comparisons and insights that were derived from the movement of sound waves were not always productive to new accounts of earthquake shock-waves, but they provided an intellectual context for the fashioning of new theories over how seismic activity operated. By focusing on the works of the Irish industrialist Robert Mallet (1810–1881), who asserted that he had made “the first attempt to bring the phenomena of the earthquake within the range of an exact science”, it becomes clear that knowledge of sound played a significant role in Victorian accounts of seismology (Mallet 1848, 51; Dean 1991, 40). Mallet was not the first to propose an explanation for the causes and effects of earthquakes. Ancient philosophers, including Aristotle, had speculated on seismic phenomena, with most accounts relating earthquakes to the weather or planetary movements. One of the few pre-nineteenth-century works to earn Mallet’s praise was Rev John Michell’s *Conjectures concerning the cause, and observations upon the phaenomena, of earthquakes* (1760), which was one of the earliest texts to describe the wave-like nature of an earthquake.^4^ But what Mallet did was to bring his experience as an engineer to the subject and apply mechanical principles to the movements of seismic waves. Sound knowledge not only provided rich analogies to promote such accounts, but helped to shape them.

## Sonorous investigations of nature

**Figure 1.**
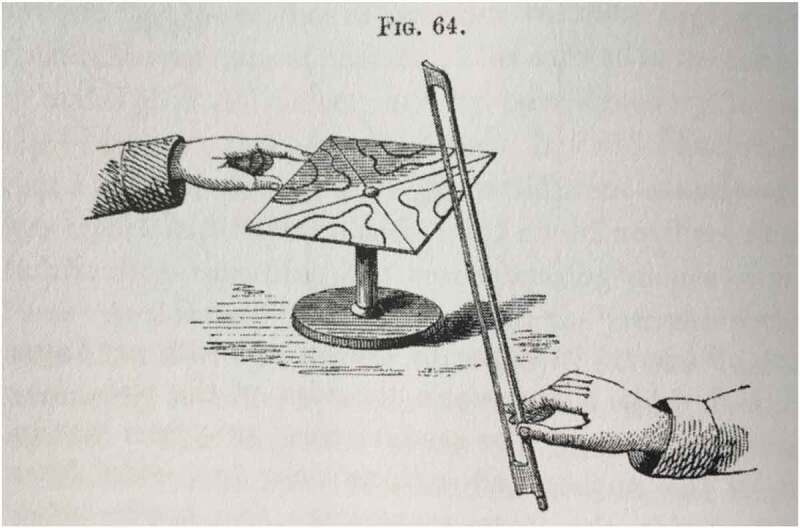
The production of an acoustic figure through Chladni’s experimental method of putting a plate covered in sand into vibration, here employing a violin bow to cause the agitation. (From Tyndall 1867, 142). Image in author’s possession, 2019).

In his 1787 *Entdeckungen über die Theorie des Klanges*, the German natural philosopher Ernst Chladni published an account of a series of experimental practices which sought to make sonorous phenomena visually observable. The basic principle of this was to take a glass plate, capable of transmitting a vibratory impulse, covering it with sand and then disturbing the plate, usually by drawing a bow along its edge. (Figure 1) This motion caused the plate to vibrate and sound a note, but at the same time the sand was agitated and convulsed into patterns which revealed the wave motions passing through the solid body. Sand assembled along nodal lines (the areas of least vibrating motion) and the patterns these formed, which became known as “Chladni figures”, varied depending on the frequency of the tone the glass plate sounded. This celebrated experimental arrangement made sonorous waves visible to the eye, as well as to the ear, and was subject to considerable philosophical interest in early-nineteenth-century Europe.^5^ French natural philosopher Felix Savart (1791–1841) and Danish chemist Hans Christian Ørsted (1777–1851) both repeated and extended Chladni’s trials, with particular attention paid to how sound and electricity might be relatable (Pesic 2014, 181–94; Faraday 1859, 315). In Britain, Charles Wheatstone (1802–1875) conducted even more extensive experiments, cataloguing a diverse range of possible patterns.^6^ In Germany, brothers Ernst Heinrich Weber (1795–1878) and Wilhelm Weber (1804–1891) performed similar experiments and adapted these by using water instead of sand to observe the motions of waves. The Webers filled a square vessel with water or mercury and stimulated two series of parallel waves to intersect with two series of long waves. They published their findings on wave phenomena in *Wellenlehre auf Experimente gegründet* in 1825, where they alleged that the surface of the fluid replicated the movements of Chladni’s glass plates.^7^

It was not just in the field of acoustic science that Chladni’s techniques provided useful methods of empirical inquiry. What Chladni’s figures effectively did was to make an invisible impulse, in this case a sonorous wave, visible. It was not long before natural philosophers applied Chladni’s methods to the study of other seemingly unobservable forces, such as heat, magnetism, and electricity. The London-based Michael Faraday (1791–1867) was the foremost of experimentalists to do this, almost certainly in an effort to better understand how to induce electricity from a combination of motion and magnetism. That electricity and magnetism could cause movement was already a known phenomenon. In April 1820 Ørsted had found that by passing a current through a wire, it was possible to get a closely-positioned magnetic needle to jump. Then in 1821, Faraday’s observation that a wire forming part of an electric current could be made to oscillate around a magnet established the principle of electromagnetic rotation (Cantor, Gooding, and James 1991, 47, 52). But following these early experiments, the belief that while electricity and magnetism could generate motion, motion and magnetism could produce electricity, remained unrealised throughout the 1820s. It was not until 1831 that Faraday finally delivered the promised phenomenon of electromagnetic induction, devising an experimental method to create electricity by the motion of a magnet. By moving a magnet through a helix of coiled copper wire, connected to a circuit, Faraday observed the generation of electricity (Martin 1932, 367; Faraday 1839, 1–41, 42–75).

**Figure 2.**
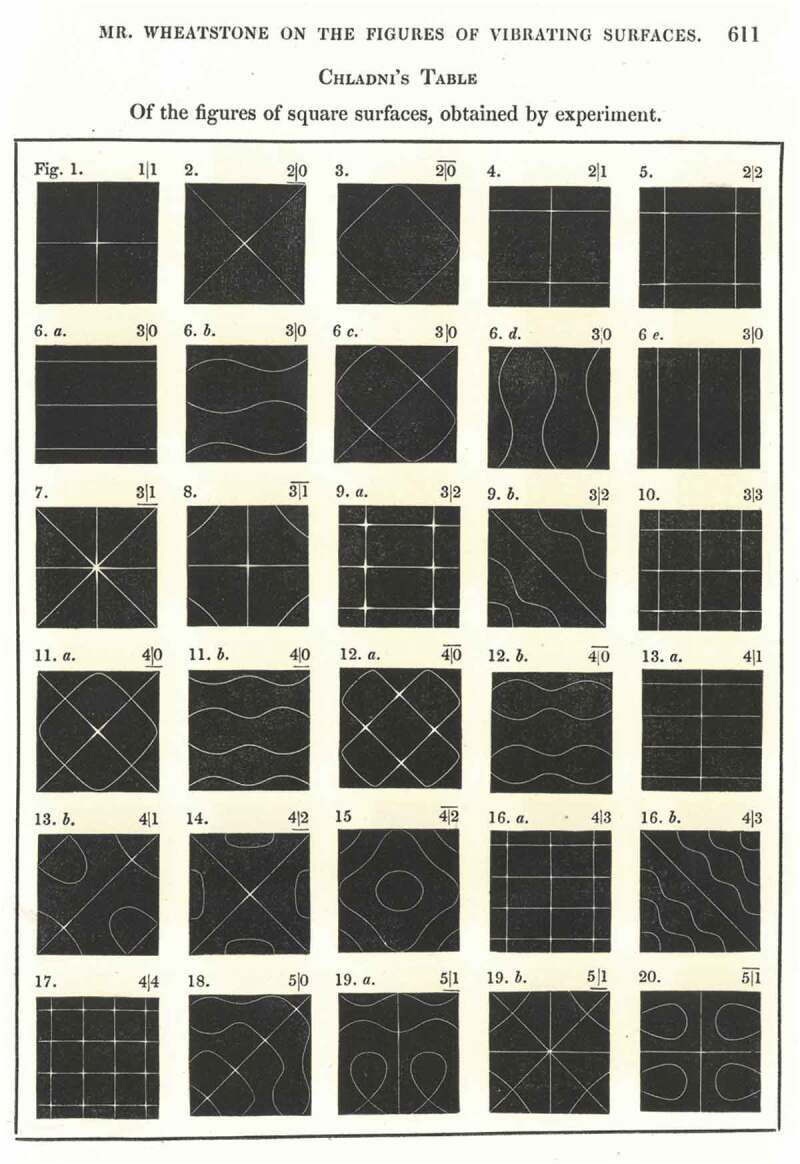
Acoustic figures from Charles Wheatstone’s experiments, published in 1833 (Image in author’s possession, 2019).

Importantly, Faraday had spent the six preceding months to conceiving of his famed electromagnetic induction arrangement working on what were effectively acoustic figures. Through his close association with Wheatstone, Faraday was already well acquainted with Chladni’s acoustic figures. Indeed, the two natural philosophers had collaborated to put on no less than 14 lectures at the Royal Institution on sonorous phenomena as part of the popular Friday Evening Discourses, held regularly in the Institution’s lecture room.^8^ Included in these were demonstrations of Chladni’s experiments, and Wheatstone published accounts of his own acoustic figure experiments in 1833 in the *Philosophical Transactions of the Royal Society of London* (Wheatstone 1879, 64–83). (Figure 2) Between February and July 1831, Faraday himself turned to the observation of vibratory waves through various media, conducting immensely thorough investigations into the phenomena. In February he began, like Wheatstone and Chladni, observing the movement of powders and grains on a vibrating body, but by the end of the month had progressed to analysing the motions of various fluids on top of vibrating surfaces: a phenomenon both he and Wheatstone termed “crispations”. For example, Faraday employed mercury on a vibrating plate, watching how the wave passing through the solid body could be examined by studying the motion of the mercury. On trying this, he found that a film developed over the mercury, so covered it in nitric acid to prevent this occurring. When he replaced the acid with ink, a particularly beautiful effect could be observed, with peaks of mercury breaking through the dark surface; this produced “the appearance of pearls of equal size beautifully arranged in a black medium” (Faraday 1859, 335, 338–9). Through such arrangements, Faraday used fluids, instead of sand, to examine the motions of waves and nodal lines that formed on a vibrating solid body.

Faraday was no doubt satisfied with the beauty of his crispation experiments; they certainly had an aesthetic value which he was keen to show off to Royal Society and Royal Institution audiences, and he soon wrote up his experimental results for the Royal Society’s *Philosophical Transactions*. However, as Peter Pesic and Ryan Tweney have both argued, the timing of these experiments seems highly relevant to Faraday’s subsequent, almost immediate, observation of electromagnetic induction.^9^ Faraday had invested considerable time and effort in attempting to produce the phenomenon during the 1820s, without success. What he recognised, within a few days of revisiting the problem on 29 August 1831, was that electricity was only caused by an impulse: it was the magnet’s movement that caused a brief electric impulse and it required repeated motions with the magnet through the coil to sustain an electric current. In this respect, the production of electricity paralleled the production of crispations; only by a continual movement, or a repetition of impulses, could waves, observed through crispation experiments, be sustained.

It is easy to see how Faraday’s efforts to render vibratory waves visible to the eye represented the adaptation of Chladni’s experimental techniques for examining sonorous impulses to the investigation of other natural forces. Yet this was just one part of a much wider dissemination of Chladni’s acoustic figures throughout European scientific and non-specialist audiences. For example, in 1835 during a lecture at King’s College London, Wheatstone instructed his listeners to go home and try Chladni’s experiments for themselves. These were experiments that were simple for all to try, regardless of social rank, education, or sex. Wheatstone offered precise instructions for creating acoustic figures

because there may be many persons present who might wish to repeat these experiments. The materials are easily to be obtained, and the great variety of figures, which with a little practise may be obtained from plates of different forms, will amply compensate for the time employed in repeating the experiments.^10^

By performing these experiments, Wheatstone’s audiences could not only enhance their understanding of sound but, supposedly, of how waves of force of all kinds operated in nature.

## Robert Mallet’s seismology

The sonorous investigation of solid bodies through Chladni’s experimental techniques not only had implications for the study of forces such as magnetism and electricity, but for geology. Indeed, the observation of the motion of sand on a vibrating glass plate was relatable to the earliest conceptions of “seismology”, including the structure of, and processes at work on, the earth’s surface. Geology was an increasingly fashionable subject in early nineteenth-century Britain, with William Smith (1769–1839) publishing his map of Britain’s geological strata in 1815 and Charles Lyell’s (1797–1875) arguments that the earth was subject to constant processes, such as erosion and volcanic activity, causing much sensation during the 1830s.^11^ However, it was the Irish industrialist Robert Mallet who first claimed to be a “seismologist”, specialising in the science of earthquakes and producing detailed accounts of how they operated. It was, after all, Mallet who first coined the word “seismology”, as well as a host of terms for the earth sciences, including “seismic” and “epicentre”.^12^ The etymology of this term, appropriately, had its origins in the Ancient Greek “seismós”, meaning “shaking”.

Born in Dublin and, after a protestant upbringing, attending Trinity College Dublin from 1826, Mallet became a partner in his father’s brass and copper business, the Victoria Foundry, during the 1830s. Profiting from the railway boom, Mallet’s company produced large amounts of iron for Ireland’s main railway lines and later secured contracts for the Guinness works in Dublin and the city’s main railway terminus (Boase 2004; Cox 1982, 1–4; Dean 1991, 39). Beyond industry, Mallet earned increasing fame for his geological work, delivering his debut paper to the Royal Irish Academy in 1837, which examined Galway’s igneous rocks. He was especially attracted to the first volume of Lyell’s *Principles of Geology* (1830) in which the geologist claimed that geological actions were governed by scientific laws, as well as by Lyell’s account of the 1783 Calabrian earthquakes (Ferrari and McConnell 2005, 46; Dean 1991, 45). Over the next 40-years, Mallet presented no less than 47 papers on the earth sciences and his work secured him a fellowship of the Royal Society in 1854 and the Geological Society of London’s prestigious Wollaston Medal in 1877. A keen supporter of the British Association for the Advancement of Science (BAAS), he attended the 1835 Dublin meeting and received funds from the association for his geological inquires between 1847 and 1860.^13^

**Figure 3.**
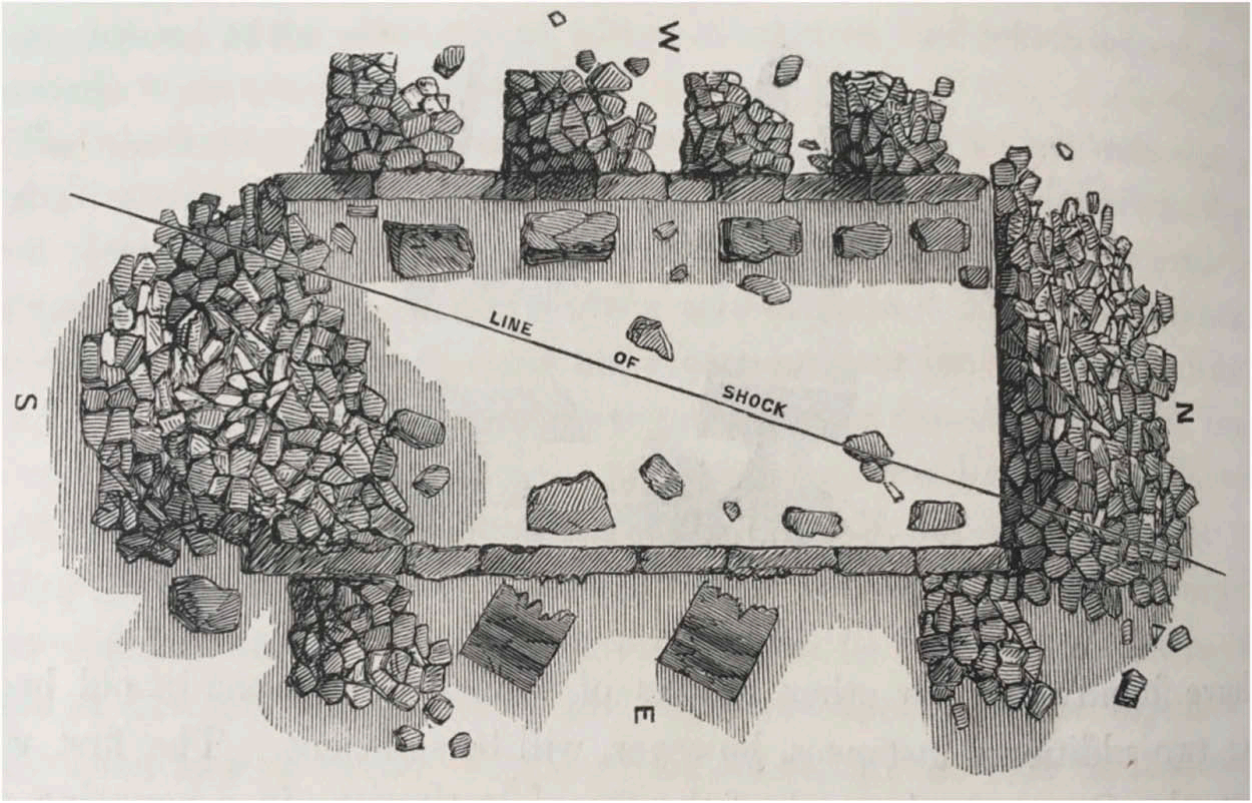
Mallet’s “line of shock” from an earthquake, as it moved through a ruined church. (From Mallet 1848, 51–105, 54). Reproduced with permission of the Institute of Astronomy Library, Cambridge, 2019).

With his experience as an engineer and industrialist, Mallet’s most prominent claim was to explain, scientifically, how earthquakes worked and, for this, the acoustic vibrations visible in Chladni’s experiments provided a valuable comparative resource. In his seminal Royal Irish Academy paper, “On the Dynamics of Earthquakes” (1846), Mallet argued that previous studies of earthquake phenomena had been poorly conceived. Instead of a vague randomly-acting force, Mallet thought of an earthquake as a single line of movement. (Figure 3) Such an impulse was, he believed, observable before society on a daily basis through the sonorous waves of music. He encouraged his readers to think of the wave of an earthquake in relation to how “the vibrations of air of a drawing-room shake the solid walls of the house, when a tune is played upon a piano-forte” (Mallet 1848, 62). Like the sonorous impulses from music played on a piano, the waves emanating from an earthquake were subject to the laws of “undulation” and Mallet explained that the “terrestrial sound wave is isochronous with the great elastic wave or earth wave of shock” which proved so destructive during seismic activity (Herries Davies 1982, 58). Citing the research of Ernst Weber on the motion of waves through water, Mallet noted that it was

not yet known that precisely analogous motions take place amongst the particles of solids within their limits of elasticity, when transmitting a wave of the first order; but it seems highly probable that there is a close analogy in the motions of the particles in both cases, so that the particles of a table of land of solid rock, transmitting an elastic wave, or earthquake shock, from a distant primary impulse, do, in all probability, describe similar epileptic curves to those of a watery wave.^14^

**Figure 4.**
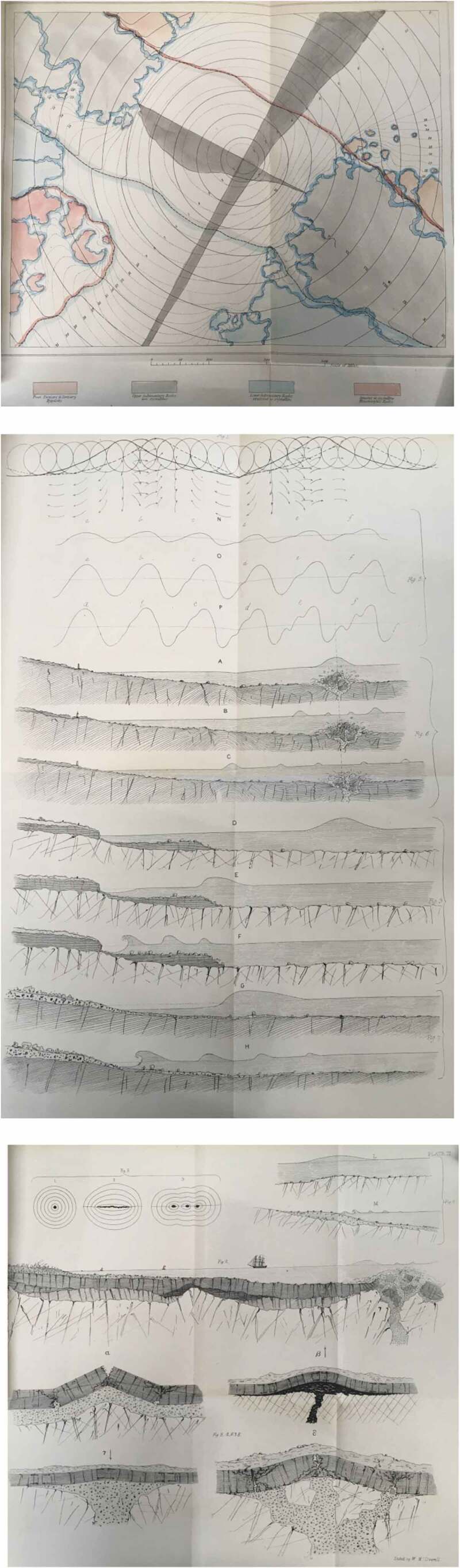
Mallet’s diagrams to show the movement of a shock wave from the epicentre of an earthquake. (From Mallet 1848, 51–105), Plates I, II, and III. Reproduced with permission of the Institute of Astronomy Library, Cambridge, 2019.

The motion of waves through water, drawing rooms, and the earth’s crust were all comparable. (Figure 4) Mallet’s intuition was correct, as it turned out, with it eventually established that seismic primary waves are longitudinal waves, exactly like sound waves, in particular, infrasound waves. From a physical point of view, the only different between sound and seismic waves is the frequent.^15^

**Figure 5.**
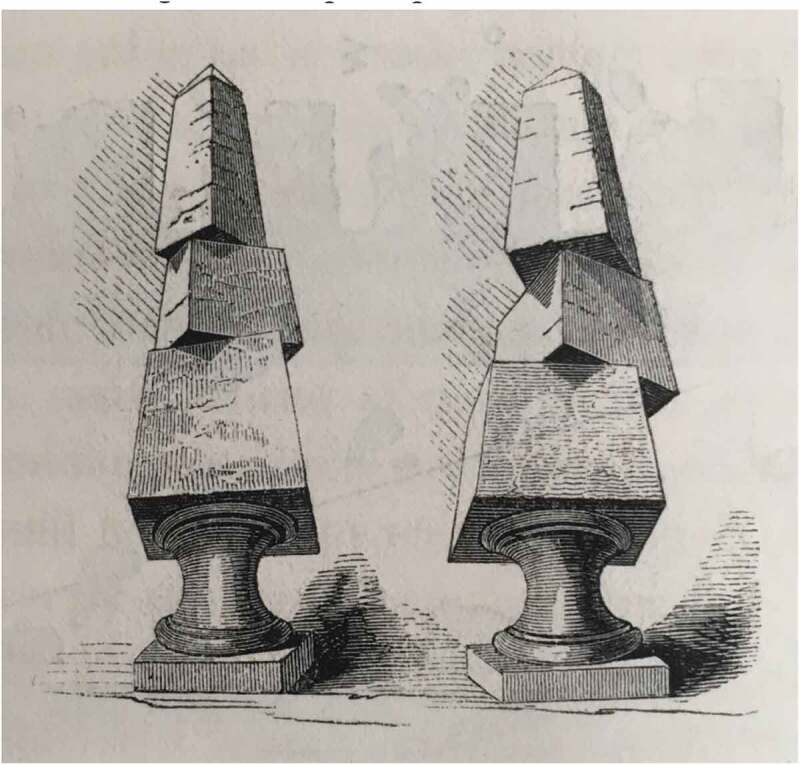
Mallet’s illustration of the manner in which earthquake shock waves caused building stones to twist, while remaining within an architectural structure. (From Mallet 1848, 51–105, 53). Reproduced with permission of the Institute of Astronomy Library, Cambridge, 2019.

Furthermore, while Weber’s work built directly on Chladni’s experiments, Mallet acknowledged that Chladni’s acoustic figures shaped his own theory on earthquakes. Yet he was careful to emphasise the limits of how far such acoustic experiments could explain seismic phenomena. Although the destructive wave of an earthquake moved in a manner similar to a sonorous vibration through a solid plate, with its speed of movement contingent on the elasticity of the earth through which it moved, Chladni’s experiments did not explain how buildings became twisted during an earthquake. Mallet observed that earthquakes produced peculiar arrangements of building stones in which they became twisted within an architectural structure but did not collapse; largely the result of a combination of primary and secondary surface waves resulting from the earthquake’s central impulse. (Figure 5) He was especially fascinated with Charles Darwin’s (1809–1882) account of an earthquake he witnessed in 1835 while in Chile during his voyage on HMS *Beagle*. Darwin recorded that the

twisting displacement … at first appears to indicate a vorticose movement *beneath each* point thus affected, but this is highly improbable … May it not … be caused by a tendency in each stone to arrange itself in some particular position, with respect to the lines of vibration, in a manner somewhat similar to pins on a sheet of paper when shaken?^16^

To this Mallet gave little credit, believing instead that “the twisting phenomenon” could be explained by the

established principles of mechanisms, without having recourse to either vortices or vibrations arranging blocks of many hundred weights after the manner of pins on paper, or sand on one of Chladni’s acoustic plates, an explanation which appears quite as far from probability as its predecessor (Mallet 1848, 55).

Mallet argued that the horizontal, as opposed to vertical, motion of a wave caused this phenomenon (Dean 1991, 45). So while the musical sounds of a piano vibrating the walls of a house could be related to the impulse of an earthquake, and the waves of a water tank or acoustic plate could be compared to the movements of the earth, Mallet stopped short of likening Chladni’s vibrating grains of sand to building stones. Nevertheless, that both he and Darwin engaged with this possibility shows that acoustic figure experiments were central to the fashioning of the new science of seismology. Such acoustic models were not just valuable analogies, but ways of theorising and working out earthquake phenomena.

Mallet was not the first to consider these links between acoustics and seismic activity. In 1833 the workman-turned-experimentalist Arthur Trevelyan (1802–1878) had claimed that his experiments, in which he used extremely hot and cold metal pokers to produce musical notes, might explain earthquake phenomena. He reflected that

Earthquakes, and the sounds accompanying them, may be caused by vibration, occasioned by heat generated far below the surface of the earth, in some enormous metallic mass, which being in contact with some cool substance, not a very good conductor of heat, the latter is violently agitated, thus producing the vibration felt in earthquakes (Trevelyan 1833, 329).

Likewise, in his lectures on natural philosophy in 1807, Thomas Young (1773–1829) speculated on the similarity of earthquake waves to sound waves, and this notion was expanded on several times in the following few decades. French chemist Joseph Louis Gay-Lussac (1778–1850) in 1823, Scottish geologist David Milne (1805–1890) in 1841, and the Prussian polymath Alexander von Humboldt (1769–1859) in 1845 all took up Young’s suggestion, but received little attention (Dean 1991, 44).

## Earthquakes and astronomy

Mallet did not just theorise on seismic activity, but performed his own experiments on the movements of terrestrial shock-waves. While Chladni, Wheatstone, Faraday, Savart, and the Webers examined the transit of vibrations through solid and fluid bodies within laboratory spaces, Mallet conducted trials on an altogether greater scale. Following his “On the Dynamics of Earthquakes”, the BAAS commissioned further investigations into earthquake phenomena, which Mallet reported to the association’s meetings in 1850 and 1854 (Dean 1991, 46, 48–9). In 1849, Mallet recorded the transit times of shock-waves passing over half a mile through the wet quartz sand of Killiney beach by exploding gunpowder beneath the earth’s surface to imitate the impulse of an earthquake. For comparison, similar trials were conducted on the granite of Dalkey Island. The shockwaves of these experiments appeared “analogous to those of earthquakes” (Mallet 1850–1853, 144). In collaboration with the director of the Armagh Astronomical Observatory, Thomas Robinson (1792–1882), he developed what he called a “seismoscope”, consisting of a mercury-filled trough which reflected a pair of cross-wires to measure minute vibrations. This was based on the reflecting mirror of an astronomical telescope for observing celestial transits. From these experiments, Mallet calculated that the vibration of a shock-wave travelled at 824.915 feet per second through wet sand (Mallet 1862a, 231). He replicated these experiments on the solid rock of the government quarries at Holyhead Mountain in Anglesey during the 1850s, using gunpowder charges of up to 12,000 lbs, largely at the expense of the Royal Society.

Again, sonorous waves offered a framework for interpretation. On presenting the results of his 1849 experiments to the Royal Irish Academy, Mallet admitted surprise at the “slowness of transit of these pulses” and attributed this to the imperfect homogeneity of the earth. Yet the slow transit times of terrestrial impulses was such that Mallet felt that, due to the “relation that his results bore to those recently obtained as to the rate of sound in wrought iron … the hitherto received theory of sounds in solids would probably require to undergo revision” (Mallet 1850–1853, 145). This comment almost certainly referred to a series of experiments Savart had conducted in 1829 and 1830 to investigate the structure of crystals and iron samples by sonorous means. By cutting iron into thin slices, covering them with sand, and then putting these into vibration, it became possible to analyse the homogeneity of this industrial product by Chaldni’s experimental methods.^17^ Mallet’s earthquake experiments represented the extension of such experimental techniques to the earth itself and united both his geological knowledge and industrial experience.

**Figure 6.**
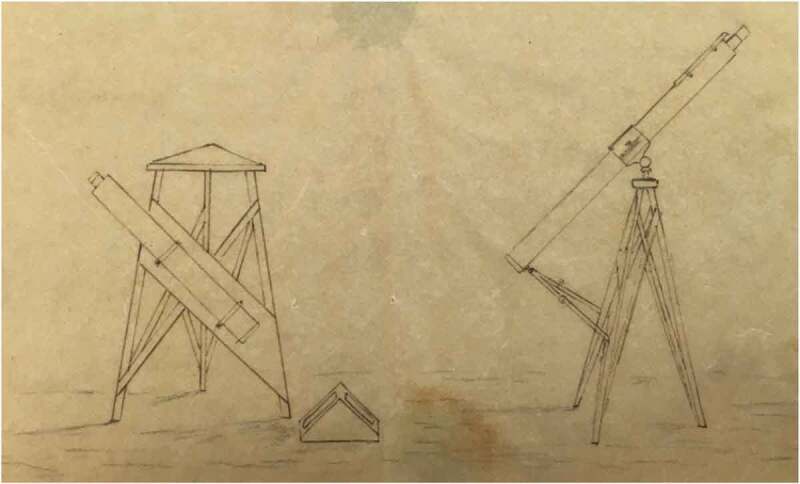
Airy’s collimater arrangement to measure vibrations from passing trains. The collimator is on the left and the telescope on the right, both angled towards the triangular trough containing mercury. (From Cambridge University Library, RGO/6/50, p. 302. Image reproduced with permission of the university library, 2019).

Mallet’s work on terrestrial impulses fit within a much broader context of interest in how vibrations travelled through the earth. It was for astronomy, a science above all about precision, that terrestrial vibrations were of most urgent concern. To take accurate observations of stellar bodies involved the use of troughs containing mercury, which reflected images of planets and stars with greater precision than could be attained directly by the eye. However, any disturbances to the trough, no matter how slight, caused tremors through the mercury which made celestial observations quite impossible.^18^ In the mid-nineteenth century the construction of railways threatened to cause just such disturbances to astronomical observatories. During the 1830s and 1840s, amid several proposals to build railway lines through Greenwich Park near the Royal Observatory, there was an imperative to determine just how much vibration passing locomotives would cause and the impact of this on astronomical mercurial troughs.^19^ In 1836 the Astronomer Royal, George Biddell Airy (1801–1892), conducted a series of experiments with this aim, placing a mercury-filled trough at various distances from passing trains. A collimator directed at the sun projected an image of the stellar body and several wires inside the device onto the mercury, and this image was then observed through a telescope focused on the trough, providing a highly magnified view to observe minute vibrations.^20^ (Figure 6) On finding that passing trains would certainly interfere with astronomical readings, threatening in particular to undermine the daily transit observations required to calculate Greenwich Time, the proposals for a park railway were shelved. However, on several occasions in the 1850s and 1860s, railway companies launched fresh bids to build a line near the Observatory; such a route would improve connections to Woolwich, Gravesend, and East Kent, while the park offered a cheap alternative to building through housing in Greenwich.^21^

In 1863 the London, Chatham, and Dover Railway Company orchestrated a particularly aggressive attempt to build a Greenwich Park route, even suggesting the relocation of the Royal Observatory away from Greenwich. Airy repeated his earlier collimator experiments, this time near the Metropolitan Railway in Regent’s Park but, in 1865, with growing pressure from the South Eastern Railway Company, sought further evidence that terrestrial tremors threatened the integrity of the Observatory’s astronomical work.^22^ He sent the Observatory’s chief assistant, Edward James Stone (1831–1897), to conduct new experiments in collaboration with Mallet, by now a leading authority on terrestrial vibrations. Stone and Mallet travelled to a bog 37 miles outside Dublin where the railway passed over sleepers resting on soft muddy ground. Stone’s task was to determine if this softer foundation could absorb some of the vibrations of passing locomotives, by employing the same experimental techniques Airy had used earlier. Stone and Mallet produced a report based on several days of trials, concluding that although the bog did diminish the effects of tremor on mercury, it was only slight. Mallet and Stone’s report upheld Airy’s continued opposition to a Greenwich Park railway.^23^

Mallet’s work on the impact of terrestrial vibrations on astronomical observations built directly on his earlier inquiries into the movement of earthquake shock-waves. But it is also clear that these mercury trough trials were closely related to the earlier crispation and acoustic figure experiments of the 1820s and 1830s, only this time, scaled up and moved beyond the laboratory. Indeed, while Mallet conceived of the earth’s surface as a ginormous vibrating plate, astronomical mercury trough experiments were similar to Faraday’s work on crispations. The transfer of techniques developed to investigate sonorous phenomena were refashioned to a range of natural inquiries, including electromagnetism and terrestrial vibration, from earthquakes to railway tremors.

## Imperial seismology

Mallet’s most direct relating of seismic and sonorous phenomena featured in John Herschel’s (1849) *Manual of Scientific Enquiry; prepared for the use of Her Majesty’s Navy: and adapted for travellers in general*. This work, which the Lords Commissioners of the Admiralty commissioned as a scientific guide for naval officers on overseas expeditions, conscripted the nation’s leading scientific authorities in each discipline to provide concise accounts of natural phenomena and instruction over how these could be observed throughout the world. With Herschel as its eminent editor, Astronomer Royal George Biddell Airy provided a chapter on “Astronomy”, Charles Darwin shared a chapter on “Geology”, Edward Sabine offered instruction on “Terrestrial Magnetism”, and William Whewell, Master of Trinity College Cambridge, delivered a lesson on “Tides”.^24^ Along with other entries, including for geography, botany, meteorology, and mineralogy, was a chapter on “Earthquake Phenomena”, which Herschel commissioned Mallet to write. It was an appropriate collaboration, given Herschel’s prominence in British acoustic science. This was a real endorsement of Mallet’s authority in the field and provided an opportunity for him to shape the collection of seismic observations from across the globe, thus mobilising Britain’s huge naval resources in the study of earthquakes.

Mallet’s description of earthquake phenomena, aimed at naval officers, confirmed sound’s central role in how he characterised seismic waves. “Whenever a blow or pressure of any sort is suddenly applied … then a *pulse* or *wave* of force, originated by such an *impulse*, is transferred, through the materials acted on, in all directions as the limits of the materials permit”, wrote Mallet, as he asserted that the transfer of this “*elastic wave*” was the continuous forward movement of molecules that comprised a vibrating body. “Ordinary sounds”, Mallet continued, “are waves of this sort in air. The shaking of the ground felt at the passage of a neighbouring railway-train is an instance of such waves in solid ground or rock. A sound heard by a person under water, or the shock felt in a boat lying near a blast exploded under water, are examples of an elastic wave in a liquid”.^25^ It was to sonorous experiences that naval officers were to look in order to understand the nature of an earthquake shock wave. The earthquake was but an elastic wave, which in general was an impulse “so comparatively small that we are only conscious of them by sounds or vibrations” (Mallet 1849, 197).

Armed with this sonorous-seismic analysis, naval officers were then taken through the various stages of an earthquake, from its central impulse to the following secondary elastic waves, and accompanying sound waves (Mallet 1849, 199–20). Mallet made it clear that an earthquake was a natural phenomenon to be experienced and recorded as much as by the ear, as with the eye. After providing detailed instruction on how to construct instruments for seismic observations, he emphasised the central role of the ear in earthquake science. Each earthquake produced a sound-wave which travelled “at the same rate as the shock, or earth-wave; it is in fact *the shock (or its fractures) heard*. Notice if the sound is heard before, along with, or after the shock is felt. An observer, putting one ear in close contact with the earth, and closing the other, will hear the sound-wave through the earth separate from that through the air. … The character and loudness of the sound through each medium, and the places in an extensive district where each was heard loudest and faintest, should be noted. The duration of the sound from first to last, through either medium, accompanying each shock, is important” (Mallet 1849, 217–8). Not only was sound central to understanding earthquakes, but to observing and recording them on a global scale. Importantly, for naval officers aspiring to secure philosophical knowledge of earthquakes, Mallet recommended Herschel’s seminal treatise, “Sound”, published in the *Encyclopaedia Metropolitana* in (Mallet 1849, 223; Herschel 1830, 747–824). Mallet’s science was built firmly on Britain’s most influential acoustic text. As the study of earthquake phenomena accompanied British imperial exploration and expansion, the ear was given a central role in the practice of seismology.

## Conclusion

Mallet continued to work on earthquake phenomena, visiting the Kingdom of Naples in 1858 to examine the damage of the large earthquake at Basilicata that had struck in late 1857. He collected substantial evidence on this catastrophic natural disaster, comparing it with the theory produced through his earlier experiments. With funding from the Royal Society, Mallet published his findings in 1862 in *Great Neapolitan earthquake of 1857: the first principles of observational seismology*. The evidence from this remained valuable throughout the twentieth century, being reprinted in 1987 and 2004.^26^ His subsequent paper, “Volcanic Energy: an attempt to develop its true origin and cosmical relations”, published in 1872, applied the thermodynamic principle of the conservation of energy to physical geology. Mallet here contended that the cooling and contracting of the earth’s shell created disruptive, seismic, movement. The vast terrestrial vibrations which earthquakes produced were the results of “cosmical mechanisms set in motion by terrestrial heat” (Mallet 1873, 149). The slow cooling of the universe was, he asserted, the cause of seismic impulses.

The influence of Chladni’s acoustic figures on the natural sciences in the early-nineteenth-century was considerable. His experimental techniques provided a way of making sonorous vibrations visible to the eye, but in doing this, Chladni provided a valuable resource for the study of nature more extensively. From electromagnetic phenomena to the motions of shock-waves emanating from earthquakes, the observation of vibratory impulses through solid bodies, as Chladni demonstrated, offered a fruitful analytical framework for natural philosophers. Through Wheatstone, Ørsted, Savart, and Faraday’s lectures and publications, Chladni’s experimental practices received much attention and were disseminated to broad audiences. In the hands of Mallet, these experiments offered new ways of thinking about the geological processes at work on the earth’s surface. In tracing Chladni’s acoustic figures from sonorous inquiry, to the analysis of natural forces, and finally to the study of earthquakes, this article has examined the evolution of set of experimental practices. Through Mallet’s work, the sciences of sound and seismology were closely connected in early-Victorian Britain.

## Notes

1. For example, see Pesic (2014, 214–215); Lockhart (2016).

2. For a history of light, see Cantor (1984); Pearce Williams (1965, 178).

3. For example, see Herschel (1830, 752-3); Somerville (1834, 146, 250–2).

4. Dean (1991, 40 and 44); on British seismology, see Musson (2013); also see, Musson (2004); on the history of seismology, see Davison (1978).

5. On Chladni see, Jackson (2006, 13–44); Tkaczyk (2015).

6. Wheatstone (1879); also see, Jackson (2016).

7. Wheatstone (1879, 73 and 81); Krehl (2009, 287); for a revealing comparison with Hermann Helmholtz and Richard Wagner’s contrasting use of the ocean to represent the motions of musical sounds, see Trippett (2013, 387–391).

8. Notes on these can be found in Royal Institution Archives (RI), MS F/4/C, ‘Friday Evenings, 1825-1829ʹ; RI MS F/4/K, ‘Friday Evenings, 1829-1831ʹ.

9. Pesic (2014, 198, 213); Tweney (1992a, 151–3); Tweney (1985, 205); Tweney (1992b, 33–6); on Faraday’s visual reasoning and electromagnetism, see Gooding (2006).

10. King’s College London Archives, Wheatstone 5, “Lecture IV” in “Various: 8 lectures on sound”, (c.1835).

11. Rudwick (2004); online edn, May 2012 [http://ezproxy.ouls.ox.ac.uk:2204/view/article/17243, accessed 23 August 2018]; Rudwick (2008, 553); Lyell (1834); on the sensation which Lyell’s work caused, see Secord (2014, 3); for examples of the radical impact of science at this time, see Secord (2000, 11–13).

12. Boase (2004); online edn, May 2007 [http://www.oxforddnb.com/view/article/17890, accessed 17 October 2018].

13. Herries Davies (1982, 35–6, 41); (Anon.), “Robert Mallet, F. R. S.”, *Nature*, 17 November 1881 (London, England), p. 59.

14. Herries Davies (1982, 62); it is worth comparing this with the work of the naval architect John Scott Russell on the movements of waves through water, see Russell (1885).

15. https://www.britannica.com/science/primary-wave; thanks to the kind reviewer at *Sound Studies* for this brilliant observation.

16. Darwin quoted in, Mallet (1848, 54–5); on Darwin’s observations of the earthquake, see White (2012); the same effect was observed on the Marcus Aurelius column in Rome, completed in 193 AD and subjected to centuries of seismic agency, as modelled in Boschi et al. (1995).

17. Savart (1829); (Anon.), ‘On the structure of metals, by Savart’ (1830).

18. *Correspondence relative to railway passing through Greenwich Park, 1834–1846*, Parliamentary Paper (PP) 1846: 375, p. 4.

19. Discussed in Gillin (forthcoming).

20. Royal Greenwich Observatory Archives, Cambridge University Library (CUL), RGO/6/47, “G. B. Airy to Charles Wood” (4 February 1836), pp. 173–6.

21. (Anon.), “London and Gravesend Railway Company”, *The Times*, (London, England), 20 November 1834; pg. 1; Issue 15,640 (Anon.), “Railways”, *The Morning Post*, (London, England), 21 May 1834; Issue 19,797; PP 1846: 375, p. 16 (Anon.), “The new projected railways”, *The Times*, (London, England), 18 January 1845; pg. 5; Issue 18,824; also see, Airy (1896, 178–9).

22. CUL RGO/6/50, “Wilfrid Airy to G. B. Airy” (28 March 1863), pp. 276–8; CUL RGO/6/50, “G. B. Airy to Alfred Austin” (28 March 1863), pp. 273–4; CUL RGO/6/50, “G. B. Airy to Clarence Paget” (24 February 1865), pp. 372–3.

23. CUL RGO/6/50, “Experiments made in Ireland, 27 May 1865 and May 28 – on the tremor produced by Railway trains, and on the effect of packing-up with bog-peat in diminishing the tremors, by E. J. Stone” (3 June 1865), pp. 469–77.

24. Herschel (1849); there is a wealth of historical literature on Victorian expedition science which is brilliantly reviewed in, Naylor and Schaffer (2019).

25. Mallet (1849, 196); this clearly invokes the Greenwich railway controversy explored in, Gillin (forthcoming).

26. Mallet (1862b); explored in Ferrari and McConnell (2005, 45–64).
